# Automatic target volume segmentation for offline adaptive head–and‐neck radiotherapy

**DOI:** 10.1002/acm2.70479

**Published:** 2026-03-12

**Authors:** Gilles Moliner, Maxime Michaud, Antoine Guerin, Rodolfe Verstraet, Charles Debrigode, Philippe Lang, Joel Greffier

**Affiliations:** ^1^ Department of Radiotherapy Oncology Division Carémeau University Hospital Nîmes France; ^2^ Medical Physics Unit Carémeau University Hospital Nîmes France; ^3^ Department of Radiology Medical Imaging Group, Nîmes, Carémeau University Hospital, Montpellier University Nîmes France

**Keywords:** adaptive radiotherapy, ART, deformable image registration, DIR

## Abstract

**Purpose:**

To assess the clinical utility of Smartfuse (Therapanacea, France), a deformable image registration (DIR) algorithm for automatic propagation of target volumes in the context of offline adaptive head‐and‐neck radiotherapy.

**Materials and methods:**

Ten patients underwent offline re‐planning during head‐and‐neck radiotherapy. Target volumes (GTV and CTV) were manually delineated by radiation oncologists (ROs) on both the initial CT (CT_i_) and one re‐planning CT (CT_R_). These manual contours were compared to those propagated by Smartfuse from CT_i_ to CT_R_. The geometric agreement between DIR‐propagated and RO‐delineated contours was assessed using Dice similarity coefficient (DSC), 95th percentile Hausdorff distance (HD_95_), and surface Dice similarity coefficient (sDSC) with 0 and 2 mm thresholds.

Dosimetric evaluation was conducted by comparing dose distributions from generated plans using automatically propagated target volumes (PTV_DIR_) with reference plans based on RO‐delineated targets (PTV_RO_). Coverage of RO‐delineated targets (GTV_RO_ + CTV_RO_ and PTV_RO_) was assessed using D95%, D50%, Dmax, and V95% ≥ 95%. Spatial dose differences were analyzed using dose difference (DD) metrics at 5% and 2% thresholds.

**Results:**

Median DSC, HD_95_, sDSC_0 mm_ and sDSC_2 mm_ were 0.86, 4.0 mm, 0.29 and 0.73, respectively. For D_95%_, median relative differences between DIR and RO plans were −0.6% for GTV_RO_ + CTV_RO_ and −2.1% for PTV_RO_ for D_95%_. All GTV_RO_ + CTV_RO_ reached V95% ≥ 95% with DIR plans, but only 61% of PTV_RO_ did. Spatial DD analysis showed median pass rates of 99.2% (DD_5%_) and 74.5% (DD_2%_) for GTV_RO_ + CTV_RO_, and 85.5% (DD_5%_) and 54.9% (DD_2%_) for PTV_RO_.

**Conclusion:**

Smartfuse may facilitate efficient propagation of target volumes in this study. However, medical review of auto‐propagated volumes remains essential, as dosimetric discrepancies may arise when relying solely on automatically generated PTV.

## 1 INTRODUCTION

Radiotherapy for head‐and‐neck is widely performed using intensity‐modulated radiation therapy, making it possible to sculpt a high dose‐volume to the target while sparing healthy tissues. However, during treatment, potential anatomical changes in patients (tumor shrinkage/growth, change in tumor position, weight loss) can significantly affect the accuracy of delivery of the calculated dose distribution to target and the sparing of organs at risk (OAR),^1^, ^2^, ^3^ thus compromising the effectiveness and safety of treatment. To take such changes into account, treatment plans may need to be adapted throughout the treatment period. Developed in the 90s, this approach is known as adaptive radiotherapy (ART).

ART may lead to a more accurate treatment but requires technical and time resources. Indeed, all the steps involved in treatment preparation such as computed tomography (CT) simulation, segmentation, dosimetry, quality assurance and verifications have to be repeated as many times as re‐planning is required.

Today, ART may be achieved “offline” with re‐planning steps done between treatment fractions or “online” with re‐planning steps done before delivering the treatment fraction.

Segmentation of OAR and target volumes may be achieved using atlas or, more recently with deep learning methods in order to reduce overall delineation time.

In the context of re‐planning, deformable image registration (DIR) may also be an interesting solution for propagating and deforming segmented volumes and, in particular, redefining the target volume from the initial CT (CT_i_) for the new anatomy of the patient displayed in the re‐planning CT (CT_R_).^4^, ^5^

There are many available DIR algorithms, including commercial, open source or homemade solutions.^4^ In these algorithms, the following components are required: a model to apply geometric transformation, a registration metric to quantify the degree of matching between the initial and re‐planned CT, and an optimizer with which it is possible to iterate and find the best result for the deformation vector field. These algorithms require an initialization step which consist of a rigid pre‐registration. This pre‐registration should be performed by the operator, in order to avoid erroneous results between two images with a substantial offset.^6^ However, this manual intervention may also influence the DIR and hence propagation of volumes.

Recently, a new commercial DIR algorithm (Smartfuse, Therapanacea, France) has been developed. In this algorithm, the initialization step may be manually carried out by the operator or may be automated based on a deep learning rigid pre‐registration layer. Smartfuse enables the user to obtain the propagation of segmented volumes from CT_i_ to CT_R_, based on the images’ deformation vector field.

In this context, the purpose of this study was to assess how Smartfuse with the automated initialization step, may facilitate re‐contouring of target volumes in offline head‐and‐neck ART.

Target volume segmentations performed by Smartfuse (DIR) were retrospectively compared with those of two radiation oncologists (ROs). Geometric analysis was used to assess similarities between DIR and RO segmented volumes. A dosimetric analysis was performed to assess the impact of variations in target volume segmentation on the dose‐volume histogram (DVH) and spatial dose distribution.

## 2 MATERIALS AND METHODS

### 2.1 Patient dataset

Ten head‐and‐neck consecutive patients were retrospectively included in this single center study (five followed by RO 1 and the other five followed by RO 2). For each patient, the CT_i_ and one CT_R_ were acquired (Table 1).

**TABLE 1:** Patient dataset. The percentage GTV_RO_ + CTV_RO_ change represents target volume changes between the initial CT (CT_i_) and the re‐planning CT (CT_R)_. For each patient, multiple GTVs and CTVs were manually delineated by the RO.

Patient	Age	Sex	Day before re‐planning	GTV_RO_ + CTV_RO_ change in % (mean ± SD)	Tumor site	Anatomical change
**1**	74	Male	22	−16.9% ± 23.7%	Larynx	Tumor shrinkage
**2**	74	Male	10	15.1% ± 13.0%	Oropharynx	Tumor growth
**3**	70	Female	5	7.4% ± 11.4%	Pharyngolarynx	Positioning
**4**	74	Male	42	3.6% ± 9.1%	Hypopharynx	Positioning
**5**	35	Male	10	9.1% ± 13.9%	Larynx	Tumor growth
**6**	53	Female	15	−9.0% ± 9.0%	Hypopharynx	Tumor shrinkage
**7**	44	Male	7	−3.5% ± 3.4%	Mobile tongue	Tumor shrinkage
**8**	63	Male	15	9.8% ± 7.4%	Oro/hypopharynx	Tumor growth
**9**	64	Male	32	−17.0% ± 12.2%	Oropharynx	Tumor shrinkage
**10**	59	Male	28	7.3% ± 36.3%	Oro/hypopharynx	Weight loss

An AIO™ board immobilization system and thermoplastic mask with a head support were used to immobilize patients. CTs were acquired on a Discovery CT 590 RT device (GE Healthcare, USA).

The slice thickness was set to 2.5 mm, and image reconstruction was performed using adaptive statistical iterative reconstruction (ASIR) with a strength setting of 20% (SS20%) and the Metal Artifact Reduction (MAR) algorithm (GE Healthcare, USA). OAR including the spinal cord, parotid glands, cochlea, esophagus, trachea, oral cavity, larynx, cerebellum, pharynx, lips, neck, brainstem, submandibular glands, and mandible were automatically delineated on both CT_i_ and CT_R_ using Annotate v1.9.1, an AI‐based segmentation module integrated into ART‐Plan^7^, ^8^ (Therapanacea, France), and subsequently reviewed and edited by RO.

Targets were manually delineated on CT_i_ and again on the single CT_R_ for each patient.

Targets included a primary gross tumor volume (GTVp) and multiple nodal GTVn. Clinical target volumes were defined separately for the primary tumor (CTV‐T) and for nodal levels (CTV‐N) by laterality at the prescribed dose levels. In total, 61 targets volumes were delineated.

RapidArc treatment plans were generated for CT_i_ and CT_R_. Plans were calculated using Eclipse v15.6 (Varian medical systems, USA) with a photon optimizer (PO) and anisotropic analytical algorithm (AAA) (version 15.6) for the Halcyon (Varian medical systems, USA) linear accelerator model. The prescribed dose was 70 Gy in 33 fractions at three dose levels: 54/56 Gy, 60/63 Gy, and 70 Gy.

### 2.2 Smartfuse

Smartfuse is an image‐registration module integrated into ART‐Plan (Therapanacea, France), designed for rigid and deformable multimodal image registration (CT, 4D‐CT, MR). The DIR process begins with a rigid‐registration which can be performed manually using landmarks, regions of interest, visual alignment or automatically using a deep learning layer that provides a global initialization. The DIR algorithm uses a spline‐based Free‐form deformation model^9^, ^10^ in which the displacement field is parameterized by a grid of control points whose displacements are interpolated using cubic B‐splines. Fixed and deformed images are compared using a multi‐metric aggregation function combining statistical and visual criteria, in order to adapt to different image characteristics (modality, intensity variation and artifacts). The optimization is solved using the FAST‐PD algorithm^11^ within a coarse‐to‐fine multiscale hierarchical scheme where the deformation control grid is progressively refined to capture large anatomical shifts first and subsequently model fine‐scale local deformations.

Moreover, this module also integrates on‐the‐fly propagation of structures using the calculated deformation field.

### 2.3 Automatic target volume segmentation and planning workflow

**FIGURE 1:**
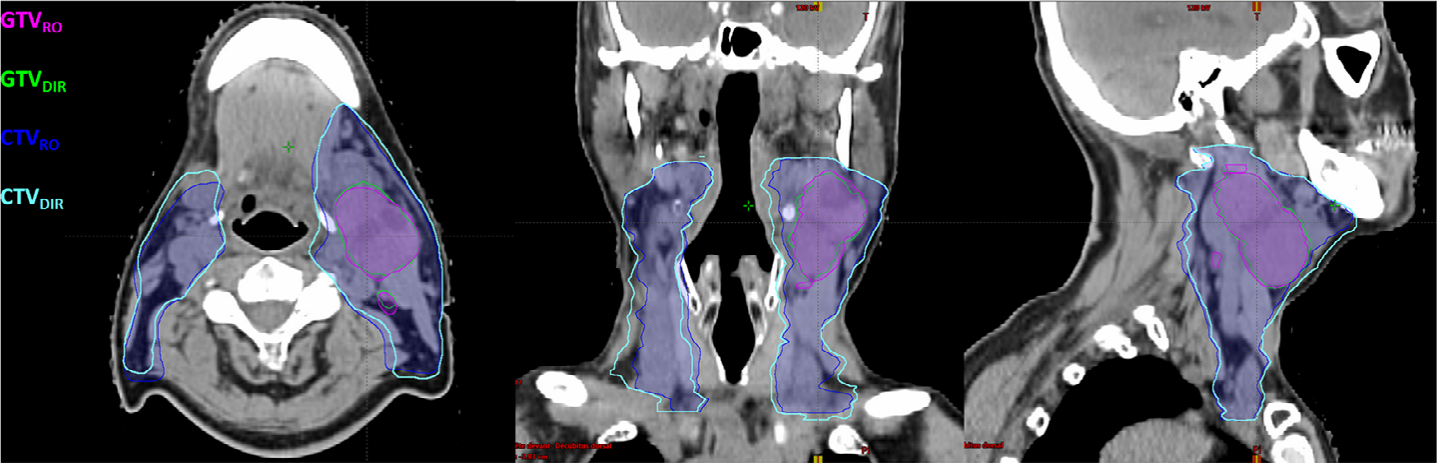
GTV and CTV manually edited by RO on replanning CT (GTV_RO_, CTV_RO_) and automatic propagation from initial CT to replanning CT using Smartfuse (GTV_DIR_, CTV_DIR_).

For each patient, a DIR of the CT_i_ to CT_R_ was made with Smartfuse in automatic mode. GTV and CTV initially delineated by the RO on CT_i_ were propagated to CT_R_ and added to the “DIR re‐planning structures set” as GTV_DIR_ and CTV_DIR_. PTV_DIR_ was generated from CTV with a 3 mm external margin. OARs delineated by Annotate and edited by RO on CT_R_ were added to the re‐planning structures set in order to achieve the optimization process. GTV, CTV and PTV delineated by RO on CT_R_ were also added to the re‐planning structures set as GTV_RO_, CTV_RO_ and PTV_RO_. (Figure 1)

A DIR re‐planning plan was generated using the PTV_DIR_ as targets and following the same optimization process (including template and normalization) as the RO re‐planning plan (which used PTV_RO_ as targets).

### 2.4 Geometric evaluation method

Geometric analysis was performed using the Dice similarity coefficient^12^ (DSC), the Hausdorff distance^13^ (HD) and the surface Dice similarity coefficient^14^, ^15^, ^16^ (sDSC) by comparing GTV_RO_ to GTV_DIR_ and CTV_RO_ to CTV_DIR_. The combined target set (GTV + CTV) was further stratified into two subsets: tumor (GTVp and CTV‐T) and node (GTVn and CTV‐N).DSC(RO,DIR) assessed the volumetric overlap between the target volume delineated by the RO on CT_R_ and the target volume propagated from CT_i_ to CT_R_ by DIR:

(1) $$\mathrm{DSC}\;\left(\mathrm{RO},\mathrm{DIR}\right)=2*\frac{V_{RO}\cap V_{DIR}}{V_{RO}+V_{DIR}}$$

where V_RO_ and V_DIR_ are, respectively, the volume in cm^3^ of target volume, delineated by the RO on the CT_R_ and propagated from CT_i_ to CT_R_ by DIR. $V_{RO}\cap V_{DIR}$ is the volume overlap between the target volume delineated by the RO and propagated by DIR. The DSC value varies from 0, meaning no overlap and 1, meaning a complete overlap. Considering DSC as an equivalent to the KAPPA index, the following analysis scale was used: A DSC value = 0 indicated no agreement and 0.01–0.20 as none‐to‐slight, 0.21–0.40 as fair, 0.41– 0.60 as moderate, 0.61–0.80 as substantial, and 0.81–1.00 as almost perfect agreement.^17^

With HD it is possible to quantify the maximum distance between the nearest points of the target volume delineated by RO on CT_R_ and propagated by DIR from CT_i_ to CT_R_. This index can be calculated as follow:

(2.1) $$HD\left(RO,DIR\right)=\max\left\{D\left(RO,DIR\right),D\left(DIR,RO\right)\right\}\;(\mathrm{mm})$$

where $RO=[ro_{1},\text{…},ro_{n}]$ and $DIR=[dir_{1},\text{…},dir_{n}]$ are the target volumes delineated by RO and propagated by DIR and composed by a number *n* of finite points.

(2.2) $$\underset{ro\in RO,\;ai\in AI}{d\left(ro,dir\right)}=\left[\left|ro-dir\right|\right]\;\left(\mathrm{mm}\right)$$

(2.3) $$D\left(RO,DIR\right)=\underset{ro\in RO,\;dir\in DIR}{\max(\min}d\left(ro,dir\right))\;\left(\mathrm{mm}\right)$$

where D(RO,DIR) represents the maximum of all the minimum distances computed for points of target volume delineated by RO and all the points of target volume propagated by DIR d(ro,dir)|ro∈RO,dir∈DIR. A similar definition is applied to D(DIR,RO). The smaller the HD, the more the volumes are similar. To avoid outliers, we decided to use the 95th percentile of HD ($HD_{95}\left(R0,DIR\right)$).


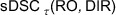
 quantifies the agreement between the surfaces of two volumes by computing the proportion of surface points that are within a certain distance threshold (τ) from each other, in both directions. In our study, we compared the surface of the target volume delineated by RO on the CT_R_ with the corresponding structure propagated from the CT_i_ by DIR.

Two thresholds were used in this study:

- τ = 0 mm, corresponding to the percentage of surface points that exactly match between the two segmentations,
- τ = 2 mm, to account for the inter‐ and intra‐operator variability reported in the literature.^18^, ^19^

The sDSC varies from 0 (no surface agreement) to 1 (perfect surface overlap), and is calculated symmetrically by taking into account both directions (RO → DIR and DIR → RO), as follows:

(3) $$\underset{ro\in RO,\;dir\in DIR}{\mathrm{sDS}C_{0mm}}=\frac{d_{\;0\;mm}\left(ro,dir\right)+d_{\;0\;mm}\left(dir,ro\right)}{d\left(ro,dir\right)+d\left(dir,ro\right)}$$

(4) $$\;\underset{ro\in RO,\;dir\in DIR}{\mathrm{sDS}C_{2mm}}=\frac{d_{\;2\;mm}\left(ro,dir\right)+d_{\;2\;mm}\left(dir,ro\right)}{d\left(ro,dir\right)+d\left(dir,ro\right)}\;$$

where $\left\{d_{0\;mm}\left(ro,dir\right),d_{0\;mm}\left(dir,ro\right)\right\}$, $\left\{d_{2\;mm}\left(ro,dir\right),d_{2\;mm}\left(dir,ro\right)\right\}$ represent, respectively, the number of points between the surfaces of RO and DIR target volumes with a distance of 0 mm and a distance of 2 mm or less.

All these analyses were performed using homemade Python scripts including libraries pydicom for dicom reading, scikit‐image for mask generation from RT structures, scipy and numpy for numerical operations.

The difference in delineation concordance between RO and DIR for node and tumor volumes was assessed by the non‐parametric Mann–Whitney U test. The Pearson correlation test was used to test relationships between GTV_RO_ + CTV_RO_ changes (Table 1) and geometric indexes in order to show whether the accuracy of the DIR process depended on the degree of modification of the target volumes. For all statistical tests, the statistical significance was set at a *p*‐value < 0.05.

### 2.5 Dosimetric evaluation method

Dosimetric evaluation of GTV_RO_, CTV_RO_ and PTV_RO_ was performed by comparing RO and DIR re‐planning plans using a DVH and spatial dose distribution analysis. Target volumes were grouped for analysis into two categories: (1) GTV_RO_ + CTV_RO_ and (2) PTV_RO_.

For the DVH analysis, the minimum doses delivered to 95% (D_95%_) and to 50% (D_50%_) of the target volume and the maximum dose (D_max_) were used. Relative differences between RO and DIR re‐planning plans were calculated.

We also analyzed whether 95% of the target volume received the 95% minimum of the prescribed dose (i.e. V_95%_ ≥ 95% of prescribed dose). Success rates were calculated for RO and DIR re‐planning plans.

Spatial dose difference between RO and DIR re‐planning dose map were studied using the dose difference (DD) method.^20^ The DD_5%_ and DD_2%_ indicators, are defined respectively as the percentage of pixels (x) within the evaluation mask of RO target structure (Ω) (GTV_RO_ + CTV_RO_ or PTV_RO_) for which dose difference Δ(x) between RO ($D_{RO}\left(x\right)$) and DIR ($D_{DIR}\left(x\right)$) pixel dose is less or equal to 5% and 2% (τ).

(5) $$\Delta \left(x\right)=\frac{\left|D_{DIR}\left(x\right)\;-\;D_{RO}\left(x\right)\right|}{D_{RO}\left(x\right)},x\;\in \Omega$$

(6) $$DD_{\tau }\left(\%\right)=100\;\times \frac{\left|x\in \;\Omega \;:\Delta \left(x\right)\leq \tau \right|}{\left|\Omega \right|}$$

All these analyses were performed using homemade Python scripts including libraries pydicom for dicom reading, scikit‐image for mask generation from RT structures, scipy and numpy for numerical operations.

As a prelude to this study, we quantified the variability in dose distribution induced solely by the treatment planning optimizer. Our reproducibility study was carried out on the same RO volumes as for the comparative study between DIR and RO. For each patient, the RO re‐planning plan was optimized 5 times using the same planner with identical objective template, optimizer/calcul parameters and normalization. DD_5%_ and DD_2%_ analyses were performed on the 10 possible comparison combinations from the 5 optimizations for each patient.

Dose distribution variability between study (DIR vs. RO) and optimizer were assessed by the non‐parametric Mann–Whitney U test. Statistical significance was set at a *p*‐value < 0.05.

## 3 RESULTS

### 3.1 Geometrical assessment

**FIGURE 2:**
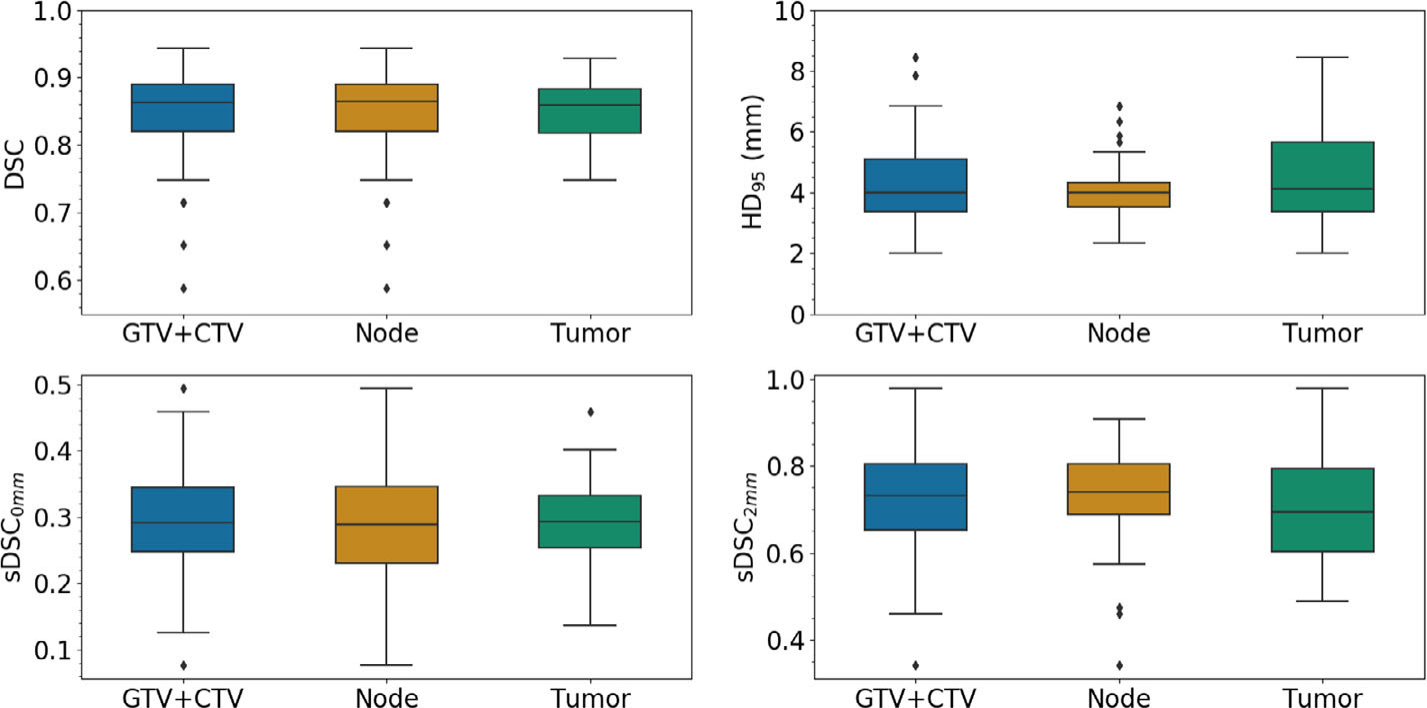
Statistical distribution of DSC, HD_95,_ sDSC_0mm_, sDSC_2mm_ comparing RO delineated targets to DIR propagated targets across 10 head‐and‐neck patients. Results are shown for the combined set GTV + CTV(*n* = 61), for tumor (GTVp + CTV‐T; *n* = 24) and node (GTVn + CTV‐N; *n* = 37). DSC, HD_95,_ sDSC_0mm_, sDSC_2mm_ are respectively the Dice similarity coefficient, 95th percentile Hausdorff distance, and surface Dice similarity coefficient with a threshold of 0 mm (sDSC_0mm_) and 2 mm (sDSC_2mm_).

**FIGURE 3:**
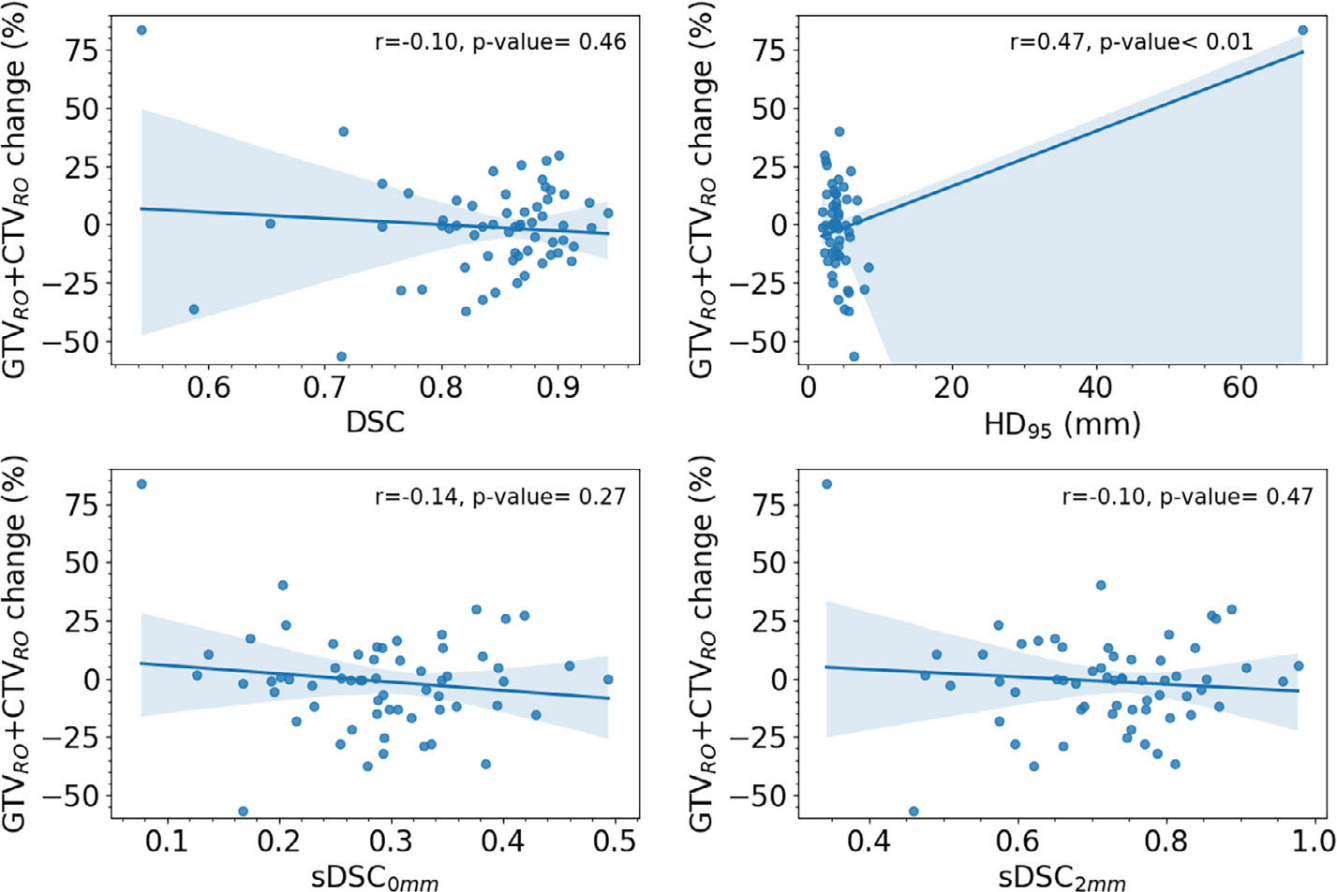
Correlation between RO target volume change from CT_i_ to CT_R_ (%) and geometric indices (DSC, HD_95,_ sDSC_0mm_, sDSC_2mm_). r corresponds to Pearson's correlation coefficient. Shaded bands indicate 95% confidence intervals. DSC, HD_95,_ sDSC_0mm_, sDSC_2mm_ are respectively the Dice similarity coefficient, 95th percentile Hausdorff distance, and surface Dice similarity coefficient with a threshold of 0 mm (sDSC_0mm_) and 2 mm (sDSC_2mm_).

The results of the geometric analysis are detailed in Figure 2 and Figure 3. Median values of DSC, HD_95_, sDSC_0mm_ and sDSC_2mm_ were respectively 0.86, 4.0 mm, 0.29 and 0.73 for the 61 GTVs and CTVs; 0.86, 4.0 mm, 0.29 and 0.74 for 37 nodes; 0.86, 4.1 mm, 0.29 and 0.69 for 24 tumors. The Mann–Whitney statistical test showed no significant difference in RO‐DIR geometric similarity metrics between tumor and node targets, suggesting similar DIR performance across target volume types (*p*‐value = 0.36 for DSC; *p*‐value = 0.27 for HD_95_; *p*‐value = 0.42 for sDSC_0mm_; *p*‐value = 0.19 for sDSC_2mm_). The Pearson's test showed a weak correlation between the group of GTV and CTV change and DSC (*r* = −0.10, *p*‐value = 0.46), sDSC_0mm_ (*r* = −0.14, *p*‐value = 0.27), sDSC_2mm_ (*r* = −0.10, *p*‐value = 0.47) and moderate correlation with HD_95_ (*r* = 0.47, *p*‐value < 0.01) due to a single outlier. This outlier (HD95 > 60 mm) represents a nodal target present on CT_R_ but not on CT_i_.

### 3.2 Dosimetric assessment

**FIGURE 4:**
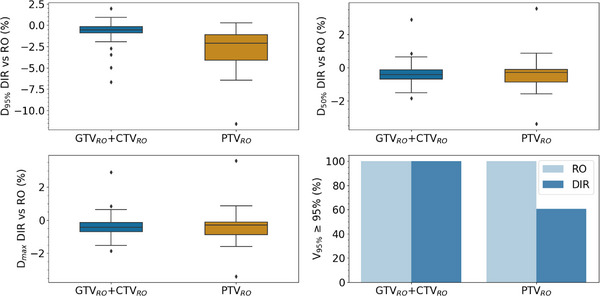
Statistical distribution of relative difference between DIR and RO plan for D_95%_, D_50%_ and V_95% ≥ 95%_ coverage dose evaluated on RO targets. Results are shown for GTV_RO_ + CTV_RO_ (*n* = 61) and PTV_RO_ (*n* = 28).

**FIGURE 5:**
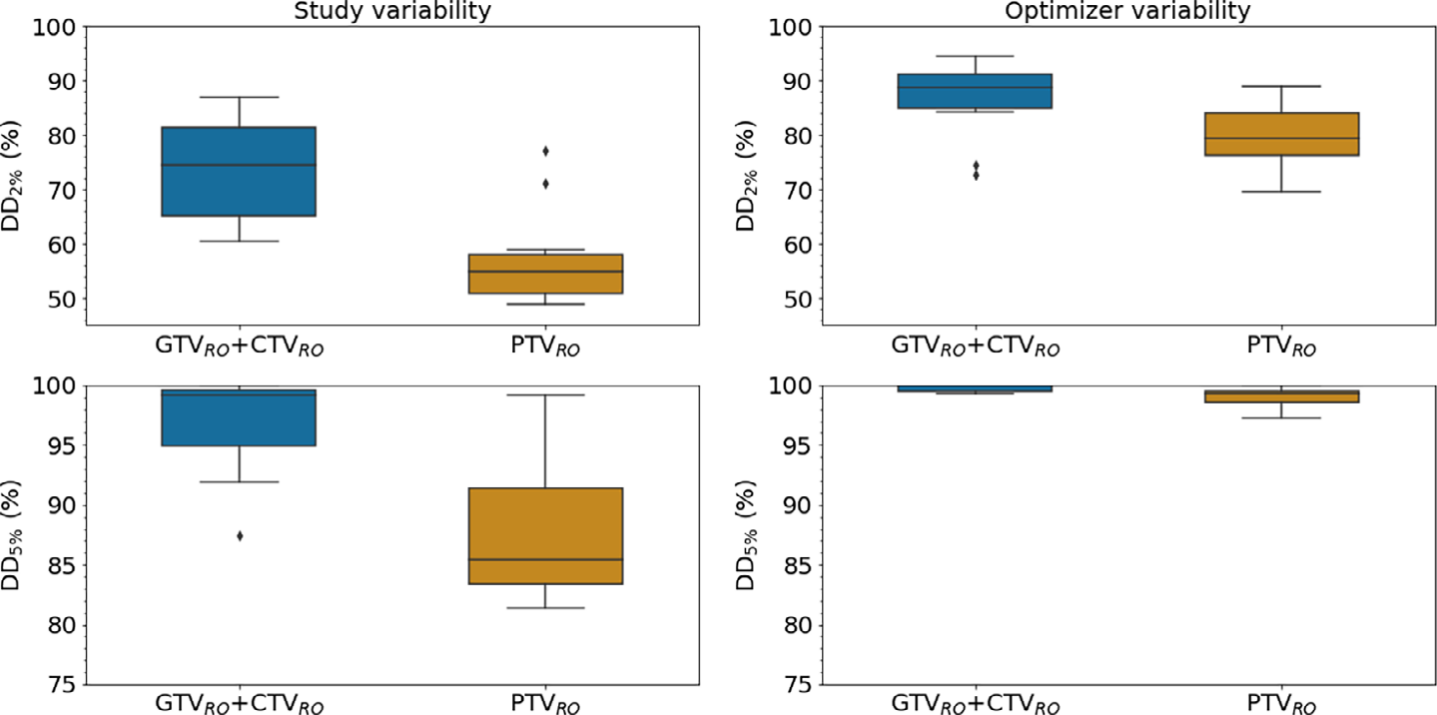
Spatial dose–difference (DD) analysis comparing (left) plan‐to‐plan variability between DIR and RO plans and (right) optimizer‐induced variability on RO plans (five re‐optimizations per patient). For each case, DD2% and DD5% pass rates were computed over GTV_RO_ + CTV_RO_ and PTV_RO_. DD2% and DD5% are respectively the percentage of pixels between RO and DIR plans with a relative dose difference of less than 2% and 5%.

The results of the dosimetric analysis are detailed in Figure 4 and Figure 5. Median values for the relative difference between RO and DIR plans on 61 GTV_RO_ + CTV_RO_ and 28 PTV_RO_ were respectively −0.6% and −2.1% for D_95%_, −0.4% and −0.3% for D_50%_, −0.4% and −0.3% for D_max_. For the GTV_RO_ + CTV_RO_ group and PTV_RO_, V95% ≥ 95% success rates were 100% for RO plans and 100% and 61%, respectively, for DIR plans. The median success rates of DD_5%_ and DD_2%_ were 99.2% and 74.5%, respectively, for GTV_RO_ + CTV_RO_; 85.5% and 54.9% for PTV_RO._ Similarly, for the optimizer reproducibility study, the median success rates of DD_5%_ and DD_2%_ were respectively 100% and 88.7% for GTV_RO_ + CTV_RO_; 99.2% and 79.5% for PTV_RO_. The Mann–Whitney statistical test showed significant difference in dose difference (5% and 2%) concordance between study and optimizer (*p*‐value = 0.05 for GTV_RO_ + CTV_RO_ (DD_5%_)_;_ *p*‐value < 0.05 for GTV_RO_ + CTV_RO_ (DD_2%_); *p*‐value < 0.05 for PTV_RO_ (DD_5%_); *p*‐value < 0.05 for PTV_RO_ (DD_2%_).

## 4 DISCUSSION

We assessed the clinical value of using the Smartfuse DIR algorithm for target volume propagation during head‐and‐neck re‐planning, based on both geometric and dosimetric analysis. The results of the usual geometric indexes, DSC (median = 0.86) and HD95 (median = 4 mm) (Figure 2), appeared to be consistent with the literature^21^, ^22^, ^23^, ^24^, ^25^, ^26^, ^27^, ^28^ (Table 2). Notably, its median DSC (0.86) is comparable to values reported for widely used tools such as SmartAdapt,^22^, ^23^ MIM,^26^, ^28^ and VelocityAI.^26^

**TABLE 2:** Literature review on the target volume deformation results of several DIR algorithms via Dice similarity coefficient (DSC) ± Hausdorff distance (HD).

Authors	Algorithms	DSC	HD (mm)
***This study***	***Smartfuse (Therapanacea)***	***Median = 0.86***	***Median HD_95_ = 4***
Zhang^24^	ANACONDA, MORFEUS (Research lab): 2 presetting parameters used	Mean ± SD for ANACONDA:0.78 ± 0.11, 0.96 ± 0.02 and MORFEUS:0.64 ± 0.15, and 0.91 ± 0.03	max 4.9
Eiland^22^	SmartAdapt(Varian medical systems)	Range GTV‐tumor: 0.54–0.88; GTV‐Nodes: 0.44–0.82	/
Hardcastle^21^	Demons, Salient‐featured‐based registration(SFBR) (Philips Healthcare)	Range for demons: 0.41–0.91 and SFBR: 0.4–0.92	Max demons: 22 and SFBR: 19
Ramadaan^23^	SmartAdapt (Varian medical systems)	Mean ± SD 0.84 ± 0.6	/
Kumarasiri^25^	B‐Spline based, demons based: 5 setup	Mean ± SD for (1):0.83 ± 0.06, (2):0.75 ± 0.15,(3):0.72 ± 0.14,(4):0.78 ± 0.09 and (5):0.68 ± 0.18	Mean for (1): 9.2,(2):7.8,(3):7.8,(4): 7.3,(5): 9
La Macchia^26^	ABBAS (Elekta), MIM (MIMVista), VelocityAI (Varian Medical Systems)	Mean ± SD for ABBAS: 0.84 ± 0.03, MIM:0.83 ± 0.03 and VelocityAI:0.81 ± 0.02	/
Tsuji^28^	MIM (MIMVista)	Mean ± SD 0.69 ± 0.12	/
Voet^27^	ABBAS (Elekta)	Range: 0.69–0.95 (operator 1) and 0.64–0.89 (operator 2)	/

We also found that the deformation of target volumes did not differ significantly between nodal and tumoral structures (Figure 2), and showed very weak correlation with the anatomical modification (Figure 3). The moderate correlation observed between HD_95_ and anatomical modification appears to be driven by an outlier case with a major anatomical difference (e.g inclusion of a new nodal disease), which may have artificially inflated the correlation coefficient. The outlier highlights an expected limitation of DIR propagation: when new nodal disease appears only on CT_R_, DIR cannot recreate it from the CT_i_ structure set. While this does not constitute formal proof of robustness, the overall lack of correlation suggests that Smartfuse's performance remains relatively stable regardless of the degree of anatomical change, supporting its potential usability across a wide range of re‐planning scenarios.

Furthermore, the geometric comparison in our study was conducted retrospectively, meaning that the RO delineations were performed entirely independently from the DIR. This avoids any potential bias introduced by the DIR and allows a more objective assessment of the algorithm's performance.

While retrospective independence is a methodological strength, it does not reflect real clinical workflows, where RO may use DIR as a starting point. In such prospective scenarios, DIR‐generated contours might help reduce inter‐operator variability as suggested by Chao et al.^29^ but also risk propagating systematic errors if not carefully reviewed.

In the geometric analysis, we also used the sDSC_0mm_ and sDSC_2mm_ to quantify the overlapping surface and also to take account of this inter‐operator variability. As expected, the sDSC_0mm_ values were lower than the DSC values, which can be explained by the known volume bias affecting DSC. sDSC provides a more stringent evaluation of boundary accuracy. By definition of the median, at least half of the targets had sDSC_0mm_ ≥ 0.29, or in other terms 29% of the DIR‐generated surface points exactly matched the RO contours. When allowing for inter‐operator variability (sDSC_2mm_), this ratio rises to 73%. These indicate that substantial portions of the surfaces required little or no adjustment.

The choice of a 2 mm sDSC cutoff for this study is a limitation, since the larger the cutoff, the greater is the ratio, but the further it is away from the RO's contour. This value was determined based on the works of Persson et al. and Brouwer et al.^18^, ^19^ who estimated inter‐operator variabilities of 2.6 and 3.9 mm respectively.

The dosimetric analysis showed no major differences in coverage of the GTV and CTV, as both structures were encompassed by the PTV margins. When evaluating the DIR‐based plans using the RO‐defined GTV_RO_ + CTV_RO_ structures, all cases achieved adequate coverage (V95% ≥ 95%). However, when assessing the RO‐defined PTV_RO_ structures on the DIR‐based plans, only 61% of the cases reached V95% ≥ 95%, indicating that the automatically generated PTV_DIR_ may fail to fully encompass PTV_RO_ in some cases. In our study, we found that, for 4 out of 10 patients, the PTV_RO_ would have been covered by the V95% whereas Voet *et al*.,^27^ reported that initial dose coverage of volumes was maintained for 1 patient out of 9.

Spatial dose‐difference analysis showed that the variation in dose distribution was greater than that linked to the optimization variability for GTV_RO_ + CTV_RO_ and PTV_RO_ when optimized on PTV_DIR_. For PTV_RO_ the median success rates of DD_5%_ and DD_2%_ fall to 85.5% and 54.9% versus 99.2% and 79.5% on optimizer variability study. Thus, even if the target volumes coverage may be acceptable (e.g., V95% ≥ 95%), the dose distribution may still differ when plans are optimized on PTV_DIR_.

Dose distribution in clinical routine is therefore impacted by the geometric difference between PTV_DIR_ and PTV_RO_. In head‐and‐neck cases, part of this discrepancy may also reflect clinical decisions, such as intentional PTV reduction to spare adjacent OAR but it may also arise from inherent limitations of DIR, which cannot recover structures or disease that are absent on the initial CT_i_.

In this work, we show Smartfuse may facilitate re‐planning workflow. The geometric agreement especially in sDSC and DSC metrics suggests that propagated contours are compatible with limited editing in some cases but this requires confirmation in prospective time‐saving studies. Nevertheless, our results suggest that automatic segmentation should always be reviewed by a RO.

It should also be remembered that this type of automatic workflow must be verified during a commissioning phase, as recommended by AAPM TG 132,^6^ so as not to bias the delineation modification by the RO.

## 5 LIMITATIONS

This single‐center, retrospective study with a small sample size (*n* = 10) limits statistical power and generalizability. No direct comparisons between multiple DIR algorithms were performed on the same dataset, so inter‐algorithm differences cannot be assessed. Our study did not objectively compare DIR segmentation variability with inter‐operator variability.

## 6 CONCLUSION

In this study, Smartfuse may facilitate re‐contouring of target volumes in context of offline head‐and‐neck ART workflow by propagating contours efficiently. However, using an auto‐generated PTV can lead to significant dosimetric discrepancies. For this reason, the target volumes should always be reviewed by a physician.

## AUTHOR CONTRIBUTIONS

**G. Moliner**: Methodology; data collection and analysis; first draft. **M. Michaud**: Methodology; data collection and analysis; first draft. **A. Guerin**: Methodology; review and editing. **R. Verstraet**: Methodology. **C. Debrigode**: Methodology. **P.Lang**: Methodology. **J. Greffier**: Methodology; review and editing. All authors have read and approved the final version of the manuscript.

## CONFLICT OF INTEREST STATEMENT

The authors declare no conflicts of interest.

## Data Availability

The datasets analyzed during the current study are available from the corresponding author upon reasonable request.
